# Four New Species and a New Combination of *Boletaceae* (*Boletales*) from Subtropical and Tropical China

**DOI:** 10.3390/jof10050348

**Published:** 2024-05-13

**Authors:** Rou Xue, Lin-Jie Su, Tai-Jie Yu, Chang Xu, Hong-Yan Huang, Nian-Kai Zeng, Guo-Li Zhang, Li-Ping Tang

**Affiliations:** 1 School of Pharmaceutical Sciences and Yunnan Key Laboratory of Pharmacology for Natural Products, Kunming Medical University, Kunming 650500, China; 18787089431@163.com (R.X.); suull121@163.com (L.-J.S.); 18385037921@163.com (T.-J.Y.); xuchang727298@163.com (C.X.); 2 Yunnan College of Modern Biomedical Industry, Kunming Medical University, Kunming 650500, China; 3 College of Medicine, Lishui University, Lishui 323000, China; hongyanyzdln@163.com; 4 College of Life Sciences, Hainan Normal University, Haikou 571158, China; niankaiz@163.com

**Keywords:** boletes, new taxa, new combination, taxonomy, molecular phylogeny

## Abstract

Previous studies have shown that boletes are abundant and diverse in China, especially in tropical and subtropical regions. In the present study, morphological, ecological, host relationship, and a four-locus (28S, *tef1*, *rpb1*, and *rpb2*) molecular phylogenetic analyses were used to study the family *Boletaceae* in subtropical and tropical China. Four new bluing species are described from three genera, viz. *Boletellus verruculosus* (Chinese name疣柄条孢牛肝菌), *Xerocomellus tenuis* (Chinese name细柄红绒盖牛肝菌), *Xer. brunneus* (Chinese name褐盖红绒盖牛肝菌), and *Xerocomus zhangii* (Chinese name张氏绒盖牛肝菌). Moreover, the genus *Nigroboletus* is treated as a synonym of *Xerocomellus*, and a new combination, namely *Xer. roseonigrescens* (Chinese name玫瑰红绒盖牛肝菌), is proposed.

## 1. Introduction

*Boletaceae* is an important group of *Boletales* (*Basidiomycota*). The majority of boletes are ectomycorrhizal fungi with favorable ecological and economic values, while a few are poisonous or bitter-tasting [1,2,3,4,5,6,7]. Besides, the taxonomy of the family *Boletaceae* has been a hot topic for scholars both in China and abroad.

*Boletaceae* was established in 1826 by the French mycologist F. F. Chevalliver. Prior to the 1980s, species identification depended on macroscopic morphological features and microscopic observations [8,9,10]. However, different scholars have given unequal weight to each characteristic, leading to continuous divergence [11,12,13,14]. In comparison to European and North American countries, early Chinese research on fungi was relatively delayed [15,16,17,18,19].

In the last two decades, enormous progress has been made in the study of boletes using the molecular criterion of polygenic genealogical concordance (GCPSR) to define species [20,21,22,23,24,25,26,27,28]. Especially in China, research on boletes has reached a climax, with the relationships between subfamilies and genera determined. Several new genera and more than 200 new species have been published [4,29,30,31,32,33,34,35,36,37,38,39,40,41,42,43].

Currently, there are about 1200 species of boletes globally, one-third of which are confirmed to be distributed in China [37]. The distribution of the family in China is territorial, mostly in subtropical and tropical areas, with obvious regional characteristics and a few species distributed across continents [4,42,44]. However, China is a vast territory with a complex and diverse geography that harbors numerous unknown fungal species. This study aims to report new bluing boletes that are distributed in the subtropical and tropical regions of China, specifically Anhui and Yunnan Provinces. Meanwhile, the system position of *Nigroboletus* Gelardi, Vizzini, E. Horak, T.H. Li & Ming Zhang is rediscussed, then a synonym of *Xerocomellus* Šutara (*Nigroboletus*) and a new combination (*Xer. roseonigrescens*) are proposed.

## 2. Materials and Methods

### 2.1. Collection Sites and Sampling

Specimens were collected from Anhui Province and Yunnan Province in eastern and southwestern China, respectively. The fresh basidiomata were recorded and photographed in the field, then dried at about 50–60 °C for 12 h. The dried specimens were deposited at the Mycological Herbarium of Kunming Medical University (MHKMU).

### 2.2. Morphological Studies

Macroscopic descriptions were based on detailed notes and photographs taken of fresh basidiomata. Color codes followed Kornerup and Wanscher [45]. For microscopic studies, 5% KOH and 1% Congo red solution (*w*/*v*) were used as mounting medium and staining agent, respectively. Microscopic structures were examined under a compound light microscope (DM2500, Leica Microsystems, Wetzlar, Germany). Basidiospores of dried specimens were examined using a ZEISS Sigma 300 scanning electron microscope (Carl Zeiss AG, Oberkochen, Germany). The following notations were used in this paper: [n/m/p] denotes ‘n’ basidiospores measured from ‘m’ basidiomata of ‘p’ collections; Q means the length/width ratio of a basidiospore in side view, and Qm is the average Q of all basidiospores ± standard deviation.

### 2.3. DNA Extraction, Amplification, and Sequencing

The procedures for DNA extraction and PCR amplification described by Tang et al. [46] were followed. The large subunit nuclear ribosomal RNA (28S), internal transcribed spacer (ITS/5.8S rRNA), translation elongation factor 1-α gene (*tef1*), RNA polymerase II largest subunit (*rpb1*), and RNA polymerase II second-largest subunit gene (*rpb2*) were amplified by polymerase chain reaction (PCR) using the primer pairs LR0R/LR5, ITS5/ITS4, EF1-983F/1567R, RPB1-B-F/RPB1-B-R, and RPB2-B-F1/RPB2-B-R, respectively [35,47,48,49]. The PCR products were checked in 1% (*w*/*v*) agarose gel, and positive reactions with a bright single band were purified and then sequenced using an ABI 3730xl DNA analyzer (Sangon Biotech, Shanghai, China) with the same primers used for PCR amplification.

### 2.4. Dataset Assembly

Fifty-nine sequences (11 of ITS, 14 of 28S, 11 of *tef1*, 10 of *rpb1*, and 13 of *rpb2*) from 14 collections were newly generated. All assembled sequences were deposited in GenBank (http://www.ncbi.nlm.nih.gov, accessed on 6 May 2024) (Table 1). Due to the high variation among species, the ITS fragment is an ideal candidate for identifying the species of most fungal groups [7,46,50,51,52,53,54,55]. However, it is usually unsuitable for building a system tree, especially at high classification levels, due to alignment difficulties. Thus, in the present analysis, ITS is excluded from the concatenated dataset (28S + *tef1* + *rpb1* + *rpb2*) of the family *Boletaceae*. Sequences for the dataset were selected from previous studies or downloaded from GenBank using the BLAST option (Table 1). Genera phylogenetically closely related to our targeted genera (*Boletellus* Murrill, *Xerocomellus*, and *Xerocomus* Quél.) in *Boletaceae* were chosen as ingroups, and generic types in *Paxillaceae* were chosen as outgroups based on the analysis of Binder and Hibbett [21]. All sequences were aligned using MUSCLE v3.6 [56] and then manually adjusted on BioEdit v7.0.9 where necessary [57]. SequenceMatrix 1.7.8 was used to concatenate the four gene fragments. The single-gene phylogenetic trees were analyzed separately and are shown in Supplementary Materials.

### 2.5. Phylogenetic Analyses

The combined dataset was analyzed using maximum likelihood (ML) and Bayesian inference (BI). Maximum likelihood tree generation and bootstrap analysis were performed using the program RAxML7.2.6, running 1000 replicates combined with an ML search [58]. Bayesian analyses were performed using MrBayes v3.2 on the CIPRES portal [59,60]. MrModeltest 2.3 was used to estimate the optimal evolution models for each subset using the Akaike information criterion (AlC) [61]. For the combined dataset, the best-fit likelihood models of 28S, *tef1*, *rpb1*, and *rpb2* were GTR + I + G, GTR + I + G, HKY + I + G, and K80 + I + G, respectively. Bayesian analysis of the combined nuclear dataset was repeated for 4 million generations and sampled at intervals of 1000. Once the average standard deviation of split frequencies went below 0.01, the run was terminated. Trees sampled from the first 25% of the generations were discarded as burn-in, and Bayesian posterior probabilities (PP) were then calculated for a majority consensus tree of the retained Bayesian trees.

**Table 1:** Species, isolates, locations, and GenBank accession numbers of the DNA sequences used in this study.

Species Name	Isolate	Locality	GenBank Accession No.					References
			28S	ITS	*tef1*	*rpb1*	*rpb2*	
*Aureoboletus catenarius*	HKAS54463	Yunnan, SW China	KT990509	—	KT990710	KT990890	KT990348	[36]
*A. catenarius*	HKAS54467 *	Yunnan, SW China	KT990510	—	KT990711	—	KT990349	[36]
*A. erythraeus*	FHMU3144 *	Hainan, S China	MT650076	—	—	MT650114	—	[43]
*A. erythraeus*	FHMU1053	Hainan, S China	MT650074	—	MT650112	—	MT650124	[43]
*A. gentilis*	MG372a	Italy	KF112344	—	KF134014	KF112557	KF112741	[35]
*A. gentilis*	Pug1	Germany	DQ534635	—	KF030399	—	—	[21]
*Boletellus ananas*	ARB1223	USA	KP327618	—	KP327671	—	—	[62]
*Bol. ananas*	NY815459	Costa Rica	JQ924336	—	KF112308	—	KF112760	[35]
*Bol. badiovinosus*	REH8923	Australia	KP327640	—	KP327693	—	—	[62]
*Bol. chrysenteroides*	BD394	USA	HQ161867	—	—	HQ161836	—	[63]
*Bol. chrysenteroides*	3838	USA	KF030312	—	KF030432	KF030383	—	[64]
*Bol. emodensis*	FHMU2031	Hainan, S China	MW826857	—	MW925890	—	—	[42]
*Bol. emodensis*	FHMU2245	Fujian, SE China	MW826863	—	MW925894	—	MW925940	[42]
*Bol. nordestinus*	UFRN Fungos2726 *	Brazil	MG760444	—	—	—	—	[65]
*Bol. putuoensis*	FHMU6907 *	Zhejiang, E China	ON166664	—	ON155822	—	ON155834	[66]
*Bol. putuoensis*	FHMU6959	Zhejiang, E China	ON166665	—	—	—	ON155835	[66]
*Bol. shoreae*	AP 6679	India	MH608211	—	—	—	—	[67]
*Bol. shoreae*	AP 6696 *	India	MH608210	—	—	—	—	[67]
*Bol. sinochrysenteroides*	FHMU3264 *	Jiangxi, E China	MW826902	—	MW925904	—	MW925947	[42]
*Bol. sinochrysenteroides*	FHMU3265	Zhejiang, E China	MW826901	—	MW925903	—	MW925946	[42]
*Bol. subglobosus*	FHMU3256 *	Hainan, S China	MW826844	—	MW925872	—	—	[42]
*Bol. subglobosus*	FHMU3257	Hainan, S China	MW826843	—	MW925874	—	—	[42]
***Bol. verruculosus***	**MHKMU H.Y. Huang 674**	**Yunnan, SW China**	**PP179428**	**PP189883**	**—**	**PP195251**	**PP195264**	**This study**
***Bol. verruculosus***	MHKMU H.Y. Huang 682 *	**Yunnan, SW China**	**PP179425**	**PP189882**	**PP230535**	**PP195249**	**PP195261**	**This study**
***Bol. verruculosus***	**MHKMU H.Y. Huang 699**	**Yunnan, SW China**	**PP179426**	**PP189881**	**PP230536**	**PP195250**	**PP195263**	**This study**
***Bol. verruculosus***	**MHKMU Y.J. Pu 216**	**Yunnan, SW China**	**PP179427**	**PP189884**	**PP230538**	**PP195248**	**PP195262**	**This study**
***Bol. verruculosus***	**MHKMU S. Jiang 414**	**Yunnan, SW China**	**PP179429**	**PP189885**	**PP230537**	**PP195247**	**PP195260**	**This study**
*Boletus edulis*	HMJAU4637	Russia	KF112455	—	KF112202	KF112586	KF112704	[36]
*B. monilifer*	HKAS83098	China	KM820807	—	—	KM820817	—	[36]
*B. monilifer*	HKAS83205	China	KM820806	—	—	KM820816	—	[36]
*B. orientialbus*	HKAS62907 *	Fujian, SE China	JN563856	—	—	JN563873	—	[36]
*B. orientialbus*	HKAS62908	Fujian, SE China	JN563857	—	—	N563874	—	[36]
*B. reticuloceps*	HKAS51232	China	KT990537	—	KT990739	KT990906	KT990376	[36]
*B. reticuloceps*	HKAS57671	China	KF112454	—	KF112201	KF112648	KF112703	[36]
*B. violaceofuscus*	HKAS62900	China	JN563859	—	KF112219	—	KF112762	[36]
*B. violaceofuscus*	HKAS62901	China	JN563860	—	—	JN563877	—	[36]
*Gyrodon lividus*	REG Gl1	Germany	—	—	GU187701	GU187461	GU187786	[68]
*Gyrodon* sp.	HKAS57588	China	KF112348	—	KF112275	KF112640	KF112817	[35]
*Gyrodon* sp.	HKAS59448	China	KF112349	—	KF112276	KF112641	KF112818	[35]
*Heimioporus gaojiaocong*	Zeng2788	Yunnan, SW China	MF962380	—	MF962410	—	—	[69]
*H. gaojiaocong*	Zeng2864	Yunnan, SW China	MF962385	—	MF962414	—	—	[69]
*H. japonicus*	HKAS52237	Yunnan, SW China	KF112347	—	KF112228	KF112618	KF112806	[35]
*H. japonicus*	HKAS80583	Yunnan, SW China	—	—	—	KT990929	KT990408	[36]
*H. subretisporus*	HKAS80581	Yunnan, SW China	KT990573	—	KT990769	—	KT990407	[36]
*H subretisporus*	HKAS80582	China	KT990574	—	KT990770	—	KT990409	[36]
*Hemileccinum impolitus*	HKAS84869	Germany	KT990575	—	KT990771	KT990930	KT990410	[36]
*Hem. impolitus*	Bim1	Germany	AF139715	—	JQ327034	KF030375	—	[20]
*Hem. rugosum*	HKAS84355 *	Yunnan, SW China	KT990578	—	KT990774	KT990931	KT990413	[36]
*Hem. rugosum*	HKAS50284	China	KT990576	—	KT990772	—	KT990411	[36]
*Hem. subglabripes*	72206	USA	KF030303	—	KF030404	KF030374	—	[64]
*Hem. subglabripes*	MICH KUO-08301402	USA	MK601739	—	MK721093	—	MK766301	[70]
*Hortiboletus amygdalinus*	HKAS54166	Yunnan, SW China	KT990581	—	KT990777	KT990933	KT990416	[36]
*Hor. amygdalinus*	HKAS54242	Yunnan, SW China	KT990580	—	KT990776	—	KT990415	[36]
*Hor. rubellus*	VDKO0403	Belgium	—	—	—	—	MH614774	[71]
*Hor. rubellus*	MICHKUO-06081002	USA	MK601741	—	MK721095	—	MK766303	[70]
*Hor. subpaludosus*	HKAS52659	Yunnan, SW China	KT990582	—	KT990778	—	KT990417	[36]
*Hor. subpaludosus*	HKAS68158	Yunnan, SW China	KT990583	—	KT990779	KT990934	KT990418	[36]
*Hourangia cheoi*	HKAS52269	Yunnan, SW China	KF112385	—	KF112286	KF112628	KF112773	[72]
*Hou. cheoi*	HKAS68306	Yunnan, SW China	KP136950	—	KP136929	KP136972	KP136980	[72]
*Hou. microcarpa*	HKAS53378	Yunnan, SW China	KF112452	—	KF112300	—	KF112775	[72]
*Hou. nigropunctata*	FHMU3113	Hainan, S China	MT650092	—	MT650121	—	—	[43]
*Hou. nigropunctata*	FHMU2981	Hainan, S China	MT650091	—	MT650120	—	—	[43]
*Hou. nigropunctata*	HKAS76657	Yunnan, SW China	KF112388	—	KF112287	KF112629	KF112774	[35]
*Imleria badia*	MB03-098a	USA	KF030355	—	KF030423	KF030393	—	[64]
*I. badia*	HKAS74714	Germany	KC215212	—	KC215242	KC215224	—	[73]
*I. obscurebrunnea*	HKAS50477	Yunnan, SW China	—	—	KC215245	KC215233	KC215241	[73]
*I. obscurebrunnea*	HKAS52557	Yunnan, SW China	KC215220	—	KC215243	KC215225	KC215234	[73]
*I. subalpina*	HKAS56375	Yunnan, SW China	KC215217	—	KC215244	KC215231	KC215240	[73]
*I. subalpina*	HKAS74712	Yunnan, SW China	KC215218	—	KC215246	KC215230	KC215239	[73]
*Paragyrodon sphaerosporus*	MB06-066	USA	GU187593	—	GU187737	—	GU187803	[68]
*Paxillus filamentosus*	Pf1	—	AF167680	—	GU187736	—	—	[74]
*Pax. obscurosporus*	Po1	Germany	—	—	KF030442	—	—	[64]
*Pax. vernalis*	AFTOL-ID 715	China	AY645059	—	DQ457629	—	—	[35]
*Phylloporus bellus*	HKAS56763	Yunnan, SW China	JQ967196	—	JQ967153	—	—	[32]
*P. bellus*	HKAS42850	Yunnan, SW China	JQ967197	—	JQ967154	—	—	[32]
*P. catenulatus*	HKAS76157 *	Bangladesh	NG_059568	—	KR094789	KR094784	—	[75]
*P. catenulatus*	HKAS76156	Bangladesh	KR094778	—	KR094788	KR094783	—	[75]
*P. pelletieri*	Pp1	Germany	AF456818	—	JQ327036	KF030390	—	[76]
*P. pelletieri*	KM128205	England	KC215221	—	—	KC215232	—	[73]
*Porphyrellus castaneus*	HKAS52554 *	Yunnan, SW China	KT990697	—	KT990883	KT991026	KT990502	[36]
*Por. castaneus*	HKAS63076	Yunnan, SW China	KT990548	—	KT990749	KT990916	KT990386	[36]
*Por. porphyrosporus*	DJM1332	USA	HQ161850	—	—	HQ161819	—	[63]
*Por. porphyrosporus*	MB97-023	Germany	DQ534643	—	GU187734	KT990933	GU187800	[21]
*Por. scrobiculatus*	HKAS53366 *	Fujian, SE China	KF112480	—	KF112241	KF112610	KF112716	[36]
*Xerocomellus bolinii*	JAB_95	USA	MW662589	—	MW737491	MW737511	MW737472	[77]
*Xer. bolinii*	JAB_43 *	USA	MW662587	—	—	MW737509	—	[77]
*Xer. brunneus*	HKAS56311	China	KF112340	—	KF112170	KF112524	KF112684	[36]
***Xer. brunneus***	MHKMU L.P. Tang 3774 *	**Yunnan, SW China**	**PP179422**	**PP189878**	**PP230532**	**PP195246**	**PP195257**	**This study**
*Xer. chrysenteron*	Xch1	—	AF050647	—	KF030415	KF030365	—	[36]
*Xer. chrysenteron*	HKAS56494	Germany	KF112357	—	KF112172	KF112526	KF112685	[35]
*Xer. communis*	HKAS50467 *	Yunnan, SW China	KT990670	—	KT990858	KT991008	KT990494	[36]
*Xer. communis*	HKAS68204	Yunnan, SW China	KT990671	—	KT990859	KT991009	KT990495	[36]
*Xer. roseonigrescens*	GDGM43238 *	Guangdong, S China	NG_059586	—	KT220595	KT220591	—	[78]
*Xer. roseonigrescens*	ZT13553	Guangdong, S China	KT220589	—	KT220596	KT220592	KT220594	[78]
***Xer. tenuis***	**MHKMU L.J. Su 224**	**Anhui, E China**	**PP179419**	**PP189875**	**PP230531**	**—**	**PP195254**	**This study**
***Xer. tenuis***	**MHKMU L.J. Su 226**	**Anhui, E China**	**PP179420**	**—**	**—**	**—**	**—**	**This study**
***Xer. tenuis***	**MHKMU J. Ma 123**	**Anhui, E China**	**PP179421**	**—**	**—**	**PP195244**	**PP195255**	**This study**
***Xer. tenuis***	**MHKMU R. Xue 94**	**Anhui, E China**	**PP179416**	**PP189877**	**PP230530**	**PP195242**	**PP195252**	**This study**
***Xer. tenuis***	**MHKMU R. Xue 95**	**Anhui, E China**	**PP179417**	**—**	**PP230528**	**PP195243**	**PP195253**	**This study**
***Xer. tenuis***	MHKMU R. Xue 100 *	**Anhui, E China**	**PP179418**	**PP189876**	**PP230529**	**PP195245**	**PP195256**	**This study**
*Xerocomus fraternus*	HKAS55328 *	Yunnan, SW China	KT990681	—	KT990869	—	KT990497	[36]
*X. fraternus*	HKAS69291	China	KT990683	—	KT990871	—	—	[36]
*X. fulvipes*	HKAS52556	Yunnan, SW China	KT990672	—	KT990860	KT991010	—	[36]
*X. fuscatus*	HKAS54753	Yunnan, SW China	KT990680	—	KT990868	KT991016	—	[36]
*X. fuscatus*	HKAS53374	Fujian, SE China	KT990679	—	KT990867	KT991015	—	[36]
*X. galbanus*	HKAS76666	Henan, C China	KF112390	—	KF112292	KF112631	KF112789	[36]
*X. galbanus*	BJTC FM1790 *	Shanxi, N China	OR655217	—	OR660016	—	OR659968	[79]
*X. magniporus*	HKAS58000	China	KF112392	—	KF112293	KF112632	KF112781	[35]
*X. rugosellus*	HKAS68292	Yunnan, SW China	KT990686	—	KT990873	KT991019	—	[36]
*X. rugosellus*	HKAS58865	China	KF112389	—	KF112294	KF112630	KF112784	[35]
*Xerocomus* sp.	HKAS76853	Guizhou, SW China	KF112394	—	KF112296	KF112635	KF112783	[35]
*X. subsplendidus*	HFJAU12009	Jiangxi, E China	OQ146969	—	OQ162212	—	—	[80]
*X. subsplendidus*	HFJAU12010 *	Jiangxi, E China	OQ146970	—	OQ162213	—	—	[80]
*X. subtomentosus*	KM168813	England	KC215223	—	KC215249	—	—	[73]
*X. subtomentosus*	Xs1	Germany	AF139716	—	JQ327035	KF030391	—	[20]
*X. velutinus*	HKAS68135 *	Yunnan, SW China	KT990673	—	KT990861	KT991011	—	[36]
*X. velutinus*	HKAS52575	Yunnan, SW China	KF112393	—	KF112295	KF112633	KF112782	[36]
***X. zhangii***	MHKMU L.J. Su 225 *	**Anhui, E China**	**PP179423**	**PP189879**	**PP230533**	**—**	**PP195258**	**This study**
***X. zhangii***	**MHKMU L.J. Su 225-1**	**Anhui, E China**	**PP179424**	**PP189880**	**PP230534**	**—**	**PP195259**	**This study**

Notes: C = Central, E = Eastern, N = Northern, S = Southern, SE = Southeastern, SW = Southwestern; * holotype; newly obtained sequences are in boldface.

## 3. Results

### 3.1. Molecular Data

**Figure 1:**
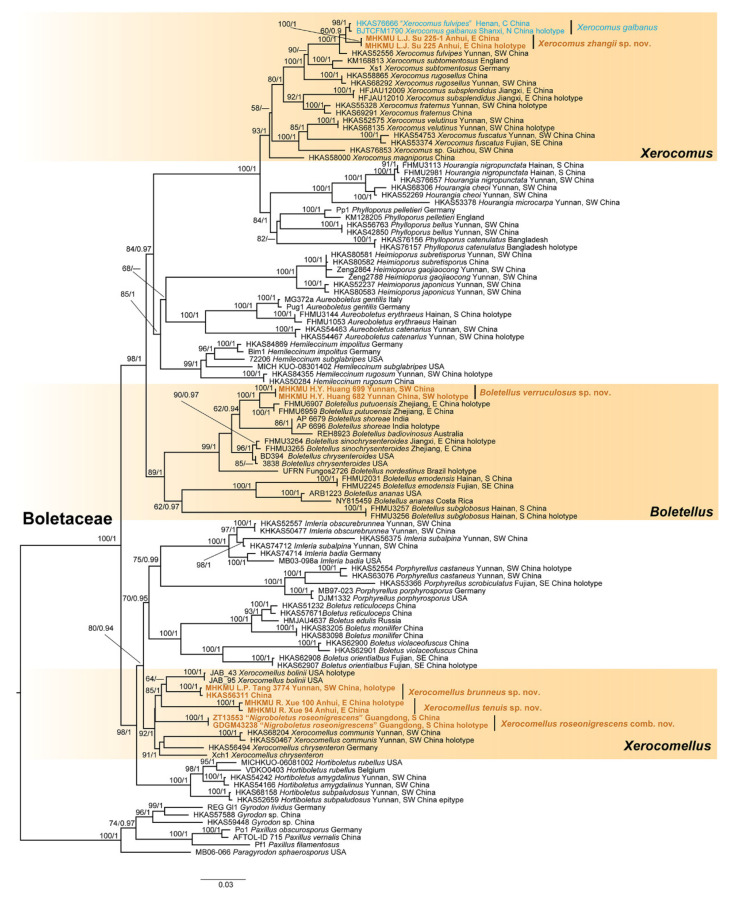
The phylogenetic tree of *Boletaceae* is based on a four-locus dataset (28S, *tef1*, *rpb1*, and *rpb2*). RAxML BP values (≥50%) and Bayesian posterior probabilities (≥0.90) are shown above the branches. Notes: C = Central, E = Eastern, N = Northern, S = Southern, SE = Southeastern, SW = Southwestern.

The combined dataset (28S + *tef1* + *rpb1* + *rpb2*) includes 112 taxa with 3601 nucleotide sites, and the alignment is available at TreeBase (Accession 31120). The tree topologies generated by the BI and ML analyses are almost identical, while the statistical support for certain relationships is slightly different. The ML tree inferred using RAxML is shown, together with the support values (Figure 1). On the basis of the molecular tree, our specimens form species-level lineages, which belong to three genera.

In the *Boletellus* clade, the first new species, *Bol. verruculosus*, is strongly supported (BS = 100, PP = 1) as an independent branch, with *Bol. putuoensis* N.K. Zeng, Yi Li, Chang Xu, Xu Zhang & J.R. Wang as its sister group (BS = 100, PP = 1). In the *Xerocomellus* group, our specimens form two lineages. The second new taxon, *Xer. brunneus*, including HKAS56311 and our collection (MHKMU L.P. Tang 3774), forms a separate branch with strong statistical support (BS = 100, PP = 1), and clusters as a sister clade to *Xer. bolinii* J.A. Bolin, A.E. Bessette, A.R. Bessette, L.V. Kudzma, J.L. Frank & A. Farid with weakly statistical support (BS = 64). The third new taxon, *Xer. tenuis*, including two collections (MHKMU R. Xue 100 and MHKMU R. Xue 94), is strongly supported as an independent branch with strong statistical support (BS = 100, PP = 1) (Figure 1). Unexpectedly, the type species of the genus *Nigroboletus* forms a lineage within the species of *Xerocomus*, with strong statistical support (BS = 100, PP = 1). In the *Xerocomus* clade, the fourth new taxon, *X. zhangii*, including two collections (MHKMU L.J. Su 225 and 225-1), forms a separate branch with strong statistical support (BS = 100, PP = 1) and clusters with *X. fulvipes* Xue T. Zhu & Zhu L. Yang and *X. galbanus* L. Fan, N. Mao & T.Y. Zhao (BS = 100, PP = 1).

### 3.2. Taxonomy

*Boletellus* Murrill, Mycologia 1: 9. 1909.
*Boletellus,* typified by *Bol. ananas* (M.A. Curtis) Murrill, is characterized by pileus covered with erect, conical, or appressed scales, yellow tined hymenophore usually turning blue immediately or sometimes unchanging when injured, and striate basidiospores with or without cross-striations on ridges [36,42,62].

**Figure 2:**
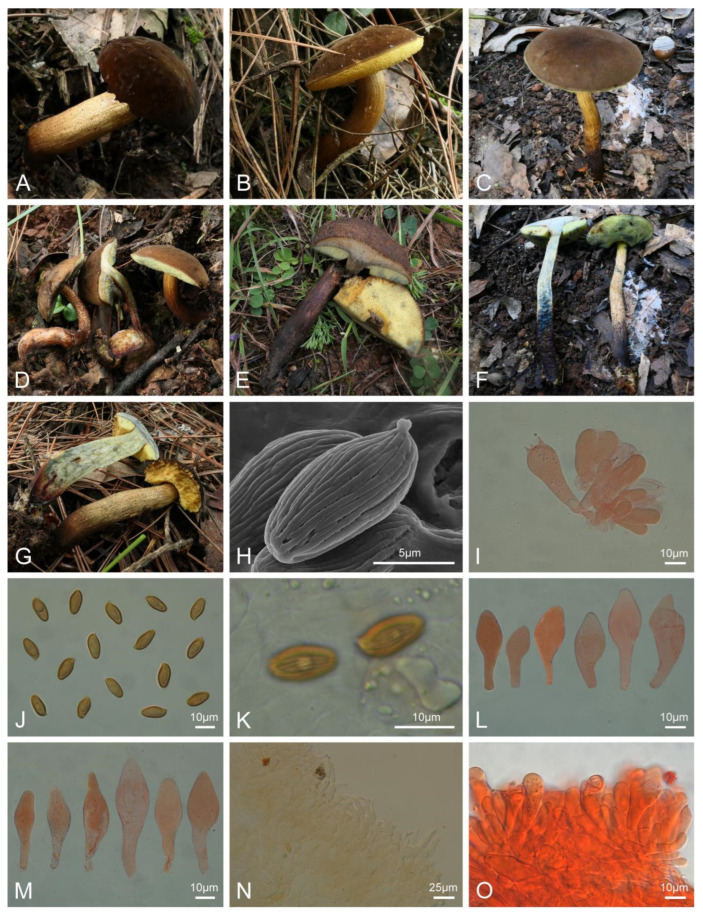
Basidiomata and microscopic features of *Boletellus verruculosus* (MHKMU H.Y. Huang 682, holotype). (**A**,**B**,**G**) MHKMU H.Y. Huang 682. (**C**,**F**) MHKMU S. Jiang 414. (**D**) MHKMU H.Y. Huang 674**.** (**E**) MHKMU Y.J. Pu 216. (**H**) Basidiospores under SEM. (**I**) Basidiomata. (**J**–**K**) Basidiospores. (**L**) Cheilocystidia. (**M**) Pleurocystidia. (**N**) Pileipellis. (**O**) Stipitipellis. (**A**,**B**,**D**,**G**) Photos by H.Y. Huang; (**C**,**F**) photos by S. Jiang; E photos by Y.J. Pu; (**H**–**O**) photos by R. Xue.

***Boletellus verruculosus*** L.P. Tang & R. Xue sp. nov. (Figure 2).

MycoBank: MB 851814.

Chinese Name: 疣柄条孢牛肝菌

Etymology: Latin “*verruculosus*”, refers to the warty scales on the stipe.

Diagnosis: Differs from other species by a brown-toned pileus, the hymenophore and context turning blue and then light brown when injured, distinctly striate basidiospores without cross-striations on ridges, and a pileipellis composed of chains of subglobose to broadly subcylindrical cells up to 19.5 μm.

Typification: CHINA. Yunnan Province: Nanhua Prefecture (南华县), near the Ma’anshan Tunnel (近马鞍山隧道), elev. 2150 m, 3 August 2020, H.Y. Huang 682 (MHKMU H.Y. Huang 682, holotype). GenBank: 28S = PP179425; ITS = PP189882; *tef1* = PP230535; *rpb1* = PP195249; *rpb2* = PP195261.

*Basidiomata* small-sized. *Pileus* 3–5 cm diam., convex to subhemispherical, margin uneven; surface dry, tomentose, velvety, brown (6D3, 6D6), cocoa brown (6E4–6E6) to dark brown (6F8); context about 0.4–0.8 cm thick, yellowish white (1A2), turning blue, then changing brown when injured. *Hymenophore* poroid, depressed around apex of stipe; pores angular to subround, 0.1 cm diam., yellow (1A4, 2A4) to pale yellowish green (29A6), turning blue, then changing brown when injured; tubes 0.4–1.1 cm in length, concolorous with pores, turning blue, then changing brown when injured. *Stipe* 5.5–8 × 0.5–0.9 cm, central, subcylindrical, solid, flexuous, sometimes enlarged at base; surface dry, yellowish white (3A2), caramel brown (6C6), grayish orange (6B5) to violet brown (10F6), covered with longitudinal stripes and cognac (6E7) to raw umber (5F8) warty scales, sometimes with yellowish brown (5E8–5F8) droplets at the base; context yellowish white (1A2) to yellow (2A6), reddish brown (8D6) to dull violet (15E4) at the base, turning blue, then changing light brown when injured; annulus absent; basal mycelium yellowish white (1A2) to sand (4B3). *Odor* indistinct.

*Basidia* 29.5–43.5 × 12–16 μm, clavate, thin-walled, 4-spored, colorless, light yellow to yellowish brown in KOH; sterigmata 3.5–6 μm in length. *Basidiospores* [100/5/5] (10–)10.5–14.5(–16) × (5–)5.5–7 μm, Q = (1.67–)1.77–2.36(–2.46), Qm = 2.05 ± 0.18, yellowish to yellowish brown in KOH, ellipsoid to subfusiform, with longitudinal or oblique ridges, 7–10 ridges visible in lateral view; ridges continuous or forked, united at the apex, projecting about 0.5 μm, without cross-striations on the ridges observed under the light microscope. *Hymenophoral trama* phylloporoid to intermediate, composed of hyphae 3.5–14.5 μm wide, colorless in KOH. *Cheilocystidia* 29–49 × 9.5–16.5 μm, abundant, subfusiform, fusiform, or subclavate, thin-walled, colorless to yellow in KOH, no encrustations. *Pleurocystidia* 32–58 × 10–18 μm, abundant, subfusiform or fusiform, thin-walled, colorless to yellow in KOH, no encrustations. *Pileipellis* epithelioid type, about 330 μm in thickness, composed of chains of subglobose to broadly subcylindrical cells up to 19.5 μm in width arising from filamentous hyphae, thin- to slightly thick-walled (up to 0.5 μm), light yellow to yellow in KOH; terminal cells 15–36 × 3.5–12 μm, subpyriform, clavate to subcylindrical, with obtuse apex. *Pileus trama* composed of interlaced, occasionally branched filamentous hyphae 5–15 μm diam., subcylindrical, thin-walled, yellowish in KOH. *Stipitipellis* hymeniform, about 100 μm in thickness, terminal cells 20.5–52.5 × 6–17.5 μm, subclavate, subfusiform or subcylindrical, light yellow to yellow in KOH, and usually with clavate, four-spored basidia. *Stipe trama* composed of longitudinally arranged, parallel hyphae 3–12.5 μm diam., cylindrical, thin- to slightly thick-walled, colorless in KOH. *Clamp connections* absent in all tissues.

Habitat: Solitary on the ground in mixed coniferous forests dominated by *Pinus yunnanensis* Franch., *Pin. armandii* Franch., including Fagaceae and Ericaceae.

Known distribution: Currently only known in Yunnan Province (elevation 2000–2400 m), southwestern China.

Additional specimens examined: CHINA. Yunnan Province: Jianchuan Prefecture (剑川县), Shaxi Town (沙溪镇), elev. 2400 m, 15 September 2019, Y.J. Pu 216 (MHKMU Y.J. Pu 216); Nanhua Prefecture (南华县), Longchuan Town (龙川镇), elev. 2130 m, 2 August 2020, H.Y. Huang 674 (MHKMU H.Y. Huang 674); Chuxiong city (楚雄市), Zixi Mountain Scenic Area (紫溪山风景区), elev. 2170 m, 4 August 2020, H.Y. Huang 699 (MHKMU H.Y. Huang 699); Qujing City (曲靖市), Dadidishui Forest Farm (大滴滴水林场), elev. 2050 m, 3 October 2021, S. Jiang 414 (MHKMU S. Jiang 414).

Notes: *Boletellus verruculosus* is both morphologically similar and molecularly related to *Bol. putuoensis*. However, the latter has cyanescent context and hymenophore, smaller basidiospores measuring 8.5–11 × 4–5 μm, and a pileipellis composed of filamentous hyphae (6–12 µm) [66].

Morphologically, *Boletellus verruculosus* is similar to *Bol. badiovinosus* E. Horak and *Bol. shoreae* A. Parihar, K. Das & Vizzini. due to the brown-toned pileus. However, *Bol. badiovinosus* has cyanescent context and tubes, relatively shorter basidiospores measuring 10−12 × 5−7 μm, and a distribution in Papua New Guinea [81]; *Bol. shoreae* has a brownish red to reddish brown pileus, cyanescent hymenophore, shorter basidiospores measuring 8–11 × 5.3–7.6 μm, and a distribution in India [67].

*Boletellus verruculosus* is also easily confused with *Bol. chrysenteroides* (Snell) Snell, *Bol. nordestinus* A.C. Magnago, *Bol. pseudochrysenteroides* A.H. Sm. & Thiers, and *Bol. sinochrysenteroides* N.K. Zeng, R. Xue & Kuan Zhao. However, *Bol. chrysenteroides* is different in its cyanescent tubes, larger basidiospores measuring 12−16 × 4.6−7.5 μm, and a distribution in North America [82]; *Bol. pseudochrysenteroides* is different in its larger basidioma, dark rose red pileus, and a distribution in North America [82]; *Bol. sinochrysenteroides* is different in its cyanescent hymenophore and context, with larger basidiospores with cross-striations on ridges (11.5−15.5 × 6.5−8 μm) [42]; *Bol. nordestinus* differs from other species in its non-cyanescent context and tubes, shorter basidiospores measuring 8−10 × 6−7 μm, and a distribution in South America [65].

*Xerocomellus* Šutara, Czech Mycol. 60: 44. 2008.
*Xerocomellus*, typified by *Xer. chrysenteron* (Bull.) Šutara. This genus is characterized by its usually cracked, subtomentose, brown, red to purple red tined pileus, hymenophore usually turning blue distinctly or sometimes unchanging when injured, relatively large pores, smooth to ornamented basidiospores [26,36,77,83].

***Xerocomellus roseonigrescens*** (Gelardi, Vizzini, E. Horak, T.H. Li & Ming Zhang) L.P. Tang & R. Xue comb. nov.

MycoBank: MB 851818.

Chinese Name: 玫瑰红绒盖牛肝菌

Basionym*: Nigroboletus roseonigrescens* Gelardi, Vizzini, E. Horak, T.H. Li & Ming Zhang, PLOS ONE 10(8): e0134295. 2015.

Known distribution: Currently known in Guangdong and Guizhou Provinces, southern and southwestern China (elevation about 280–800 m) [78,84].

Holotype: GDGM43238 (Guangdong Province, China).

Notes*: Nigroboletus roseonigrescens* was originally proposed as the type species of the monotypic genus *Nigroboletus* [78]. However, our molecular phylogeny reveals that this species should be a member of *Xerocomellus*, although no close relatives have been found yet (Figure 1). Morphologically, *N. roseonigrescens* also shares the discoloration-prone basidiomata, velvety pileus surface, yellow-tinted pores, and context with *Xerocomellus* species. Thus, *Nigroboletus* should be treated as a synonym of the genus *Xerocomellus,* and the type species of this genus should be treated as a new combination, namely *Xer. roseonigrescens*. This taxon can be easily separated from other species in the pastel pink pileus, the dull grayish to blackish discoloration of the basidiomata tissues when injured [78].

**Figure 3:**
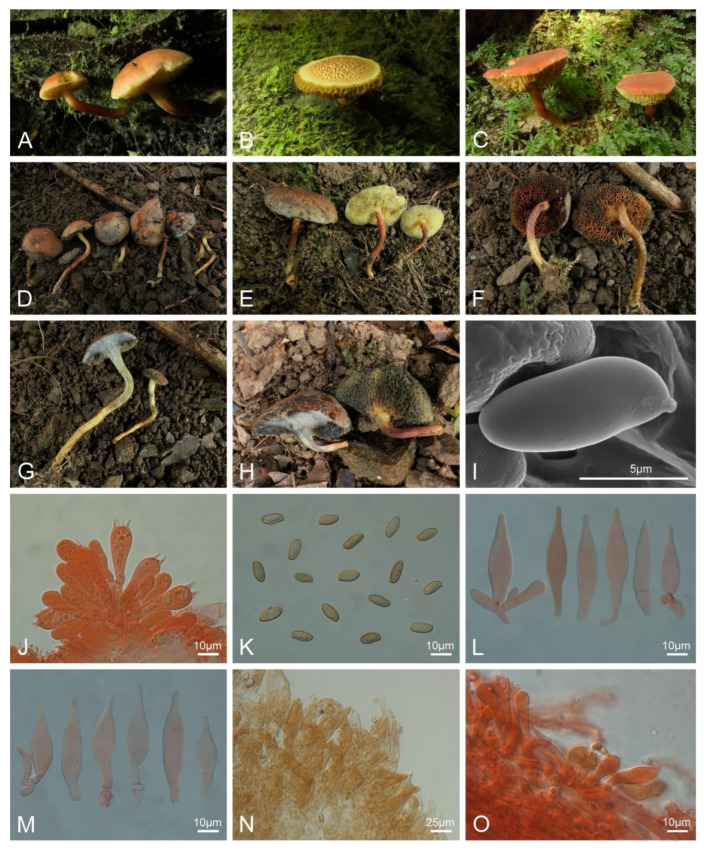
Basidiomata and microscopic features of *Xerocomellus tenuis* (MHKMU R. Xue 100, holotype). (**A**) MHKMU L.J. Su 224; (**B**) MHKMU L.J. Su 226; (**C**) MHKMU J. Ma 123; (**D**,**F**,**G**) MHKMU R. Xue 95. (**E**) MHKMU R. Xue 100; (**H**) MHKMU R. Xue 94. (**I**) Basidiospores under SEM. (**J**) Basidia. (**K**) Basidiospores. (**L**) Cheilocystidia. (**M**) Pleurocystidia. (**N**) Pileipellis. (**O**) Stipitipellis. (**A**,**B**) Photos by L.J. Su; (**C**) photos by J. Ma; (**D**–**O**) photos by R. Xue.

***Xerocomellus tenuis*** L.P. Tang & R. Xue sp. nov. (Figure 3).

MycoBank: MB 851815.

Chinese Name: 细柄红绒盖牛肝菌

Etymolog*y:* Latin “*tenuis*”, refers to the slender stipe.

Diagnosis: Differs from other species by a very small-sized basidioma, a cracked pileus covered with reddish appressed scales, a grayish yellow to brick red hymenophore, a yellowish brown to reddish brown stipe, and a pileipellis composed of inflated hyphae.

Typification: CHINA. Anhui Province: Shitai Prefecture (石台县), Qidu Town (七都镇), Huanghe Village (黄河村), elev. 160 m, 2 August 2022, R. Xue 100 (MHKMU R. Xue 100, holotype). GenBank: 28S = PP179418; ITS = PP189876; *tef1* = PP230529; *rpb1* = PP195245; *rpb2* = PP195256.

*Basidiomata* very small-sized. *Pileus* 1.8–2.7 cm diam., subhemispherical to applanate, margin decurved; surface dry, velvety, cracked, densely covered with cocoa brown (6E6), brownish orange (7C7), brick red (7D6–7), reddish brown (8E8) to dark brown (7F8) appressed scales; context about 0.2–0.4 cm thick in the center of the pileus, pale yellow (2A3), turning blue strongly and quickly, then appearing white under the cyanescence when injured. *Hymenophore* poroid, depressed around apex of stipe, occasionally 1 mm beyond the cap edge; pores angular to irregular, 0.05–0.15 cm diam., grayish yellow (2B4, 4B4), grayish orange (5B5) to brick red (7D7), turning blue strongly and quickly when injured; tubes 0.2–0.4 cm in length, concolorous with pores, turning blue strongly and quickly when injured. *Stipe* 2–3.5 × 0.2–0.4 cm, central, subcylindrical, solid, flexuous; surface dry, yellowish brown (4B5) to dead leaf (6D7), grayish red (10D5) to reddish brown (9D7), covered with weakly longitudinally stripes or brown (5D6) to dark brown (9F8) scales, pale yellow (1A2) to yellow (2A4) at apex; context pale yellow (2A3) to yellow (4A5), turning blue strongly and quickly in the upper part, brown (5D7) in the lower part when injured; annulus absent; basal mycelium yellowish white (1A2). *Odor* fresh.

*Basidia* 24–38 × 8–12 μm, clavate, thin-walled, 4-spored, colorless to yellowish in KOH; sterigmata 4–6 μm in length. *Basidiospores* [120/6/4] (9–)9.5–12(–13) × 4–5 μm, Q = (1.98–)2.1–2.75(–3.0), Qm = 2.43 ± 0.22, smooth, cylindrical, light yellow to yellowish brown in KOH. *Hymenophoral trama* phylloporoid, composed of hyphae 4.5–9 μm wide, colorless in KOH. *Cheilocystidia* 26–38 × 7.5–10 μm, abundant, subfusiform or fusiform, thin-walled, colorless to yellow in KOH, no encrustations. *Pleurocystidia* 36–62 × 8–12.5 μm, abundant, fusiform or subfusiform, thin-walled, colorless to yellow in KOH, no encrustations. *Pileipellis* palisadodermal type, about 205 μm in thickness, composed of more or less vertically arranged hyphae, expanded to 17 μm wide, occasionally 20 μm, thin- to slightly thick-walled (up to 0.5 μm), yellowish to yellowish brown in KOH; terminal cells 19–55 × 6–15 μm, subcylindrical, subconical, subpyriform to irregular, with acute apex. *Pileus trama* composed of interlaced hyphae 4–16 μm wide, subcylindrical, thin-walled, colorless to yellowish in KOH. *Stipitipellis* hymeniform, about 125 μm in thickness, terminal cells 15–50 × 4–14 μm, subclavate, subfusiform or subcylindrical, yellowish to yellowish brown in KOH, and with clavate, four-spored basidia. *Stipe trama* composed of longitudinally arranged, parallel hyphae 3–10 μm wide, cylindrical, thin-walled, light yellow in KOH. *Clamp connections* absent in all tissues.

Habitat: Solitary to gregarious on the rock moss or the ground in broad-leaved forests dominated by Fagaceae trees, e.g., *Cyclobalanopsis glauca* (Thunb.) Oerst., *Castanea seguinii* Dode, *Lithocarpus brevicaudatus* (Skan) Hay, and *Liquidambar formosana* Hance.

Known distribution: Currently only known in Anhui Province (elevation 160–200 m), eastern China.

Additional specimens examined: CHINA. Anhui Province: Shitai Prefecture (石台县), Qidu Town (七都镇), Yuantou Village (源头村), elev. 200 m, 1 August 2022, R. Xue 94, 95 (MHKMU R. Xue 94, MHKMU R. Xue 95); Qidu Town (七都镇), Xiaohekou Village (小河口村), elev. 190 m, 3 August 2022, L.J. Su 224, 226 (MHKMU L.J. Su 224, MHKMU L.J. Su 226), J. Ma 123 (MHKMU J. Ma 123).

Notes: *Xerocomellus tenuis* is molecularly related to *Xer. bolinii* and *Xer. brunneus*, while no sister taxa have been identified. However, *Xer. bolinii* has a larger basidioma, an appressed-fibrillose to squamulose pileus with pinkish brown fibrils, a pileipellis composed of filamentous hyphae, and a distribution in southeastern USA [77]; *Xer. brunneus* has a relatively larger basidioma, a rose-toned stipe, a pileipellis composed of filamentous hyphae, and a distribution in southwestern China (see below).

Morphologically, the species is similar to *Xerocomellus corneri* Xue T. Zhu & Zhu L. Yang and *Xer. carmeniae* Garza-Ocañas, J. García & de la Fuente in the size and the color of the pileus. However, *Xer. corneri* has a larger basidioma, a red brown to dull brown pileus, and a purple-toned stipe [36]; *Xer. carmeniae* has a yellowish to grayish green hymenophore, wider basidiospores measuring 10.5–13.6 × 5.7–7.8 µm, and a distribution in northeastern Mexico [83].

**Figure 4:**
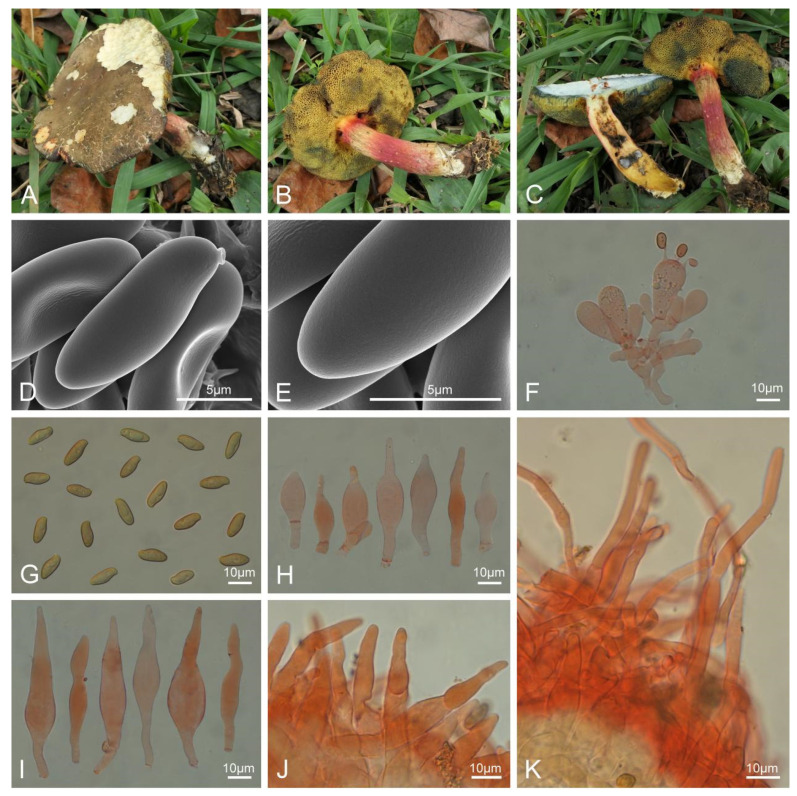
Basidiomata and microscopic features of *Xerocomellus brunneus* (MHKMU L. P. Tang 3774, holotype). (**A**–**C**) Basidiomata. (**D**–**E**) Basidiospores under SEM. (**F**) Basidia. (**G**) Basidiospores. (**H**) Cheilocystidia. (**I**) Pleurocystidia. (**J**) Pileipellis. (**K**) Stipitipellis. (**A**–**C**) Photos by L.P. Tang; (**D**–**K**) photos by R. Xue.

***Xerocomellus brunneus*** L.P. Tang & R. Xue sp. nov. (Figure 4).

MycoBank: MB 851816.

Chinese Name: 褐盖红绒盖牛肝菌

Etymology: Latin “*brunneus*”, refers to the brown pileus.

Diagnosis: Differs from other species by a cracked, velvety brown pileus, a cyanescent hymenophore and context, a rose-tinted stipe covered with longitudinal stripes, and a pileipellis composed of filamentous hyphae.

Typification: CHINA. Yunnan Province: Qujing City (曲靖市), Haizhai Forest Farm (海寨林场), elev. 2130 m, 2 October 2021, L.P. Tang 3774 (MHKMU L.P. Tang 3774, holotype). GenBank: 28S = PP179422; ITS = PP189878; *tef1* = PP230532; *rpb1* = PP195246; *rpb2* = PP195257.

*Basidiomata* small-sized. *Pileus* 5 cm diam., applanate; surface dry, velvety, cracked, olive brown (4D4), slightly reddish (9A2); context yellowish white (1A2), turning blue quickly and strongly, then appearing white under cyanescence when injured. *Hymenophore* poroid, depressed around apex of stipe; pores angular to irregular, light yellow (3A5), turning blue quickly and strongly when injured; tubes 0.5 cm in length, concolorous with pores, turning blue quickly and strongly when injured. *Stipe* 4.5 × 0.8 cm, central, subcylindrical, solid; surface dry, light yellow (3A5) to olive (3D5), titian red (7D6) to mauve (12B5), yellowish white (1A2) at base, covered with longitudinal stripes; context pale yellow (1A3), mustard yellow (3B6) at base, turning blue in the upper part when injured; annulus absent; basal mycelium grayish yellow (4B4).

*Basidia* 24–37 × 10–13.5 μm, clavate, thin-walled, 4-spored, colorless to yellowish in KOH; sterigmata 3–6 μm in length. *Basidiospores* [20/1/1] 10.5–13.5 × 4.5–5.5 μm, Q = 2.09–3.0, Qm = 2.44 ± 0.26, smooth, ellipsoid, light yellow to yellowish brown in KOH. *Hymenophoral trama* phylloporoid, composed of hyphae 3–14 μm wide, colorless to yellowish in KOH. *Cheilocystidia* 22.5–54 × 7.5–12.5 μm, abundant, subfusiform or fusiform, thin- to slightly thick-walled (up to 0.5 μm), colorless to pale yellow in KOH, no encrustations. *Pleurocystidia* 46–72 × 7.5–15.5 μm, abundant, subfusiform or subhourglass-shaped, thin- to slightly thick-walled (up to 0.5 μm), colorless to pale yellow in KOH, no encrustations. *Pileipellis* trichodermal type, about 370–450 μm in thickness, composed of filamentous hyphae 5.5–12.5 μm wide, yellowish to yellowish brown, terminal cells 18.5–42 × 4.5–11 μm, subclavate or subcylindrical, with obtuse apex. *Pileus trama* composed of interlaced hyphae 3.5–12.5 μm wide, subcylindrical, thin-walled, yellowish to yellowish brown in KOH. *Stipitipellis* a trichoderm-like structure about 60–150 μm in thickness, composed of filamentous hyphae and emergent hyphae with subclavate, subfusiform or subcylindrical terminal cells (16.5–68 × 8–16 μm), and with clavate, four-spored basidia, yellowish to yellowish brown in KOH. *Stipe trama* composed of longitudinally arranged, parallel hyphae 4.5–12 μm wide, cylindrical, thin-walled, colorless to light yellow in KOH. *Clamp connections* absent in all tissues.

Habitat: Solitary on the ground in broad-leaved forests dominated by *Cyclobalanopsis* Oerst. trees, and a small amount of *Pinus armandii*.

Known distribution: Currently only known in Yunnan Province (elevation 2130 m), southwestern China.

Notes: Molecularly, *Xerocomellus brunneus* is closely related to *Xer. bolinii* and *Xer. tenuis*. However, *Xer. bolinii* has a pinkish brown pileus and stipe and is distributed in southeastern USA [77]; *Xer. tenuis* has a very small basidioma with reddish brown-toned pileus and stipe, and a pileipellis with inflated hyphae (See above).

Morphologically, *Xerocomellus brunneus* shares a color similar to that of the pileus and stipe with three North American species, viz. *Xer. atropurpureus* J.L. Frank, N. Siegel & C.F. Schwarz, *Xer. mendocinensis* (Thiers) N. Siegel, C.F. Schwarz & J.L. Frank, and *Xer. zelleri* (Murrill) Klofac. However, *Xer. atropurpureus* is different in its purple-toned pileus, and non-cyanescent or sometimes staining blue pores [26]; *Xer. mendocinensis* is different in its gray to dark olive-brown pileus, and a stipe cover with distinct punctations [26]; *Xer. zelleri* is different in its gray to dark vinaceous black pileus, and non-cyanescent pores [26].

*Xerocomus* Quél. Fl. Vosges, Champ.: 477. 1887.
*Xerocomus*, typified by *X. subtomentosus* (L.) Quél. This genus differs from other genera in its tomentose pileus, relatively large pores, yellowish hymenophore, and context usually slightly turning blue or sometimes unchanging when injured, basidiospores with bacillate surface ornamentation, or sometimes smooth under SEM [36,42].

**Figure 5:**
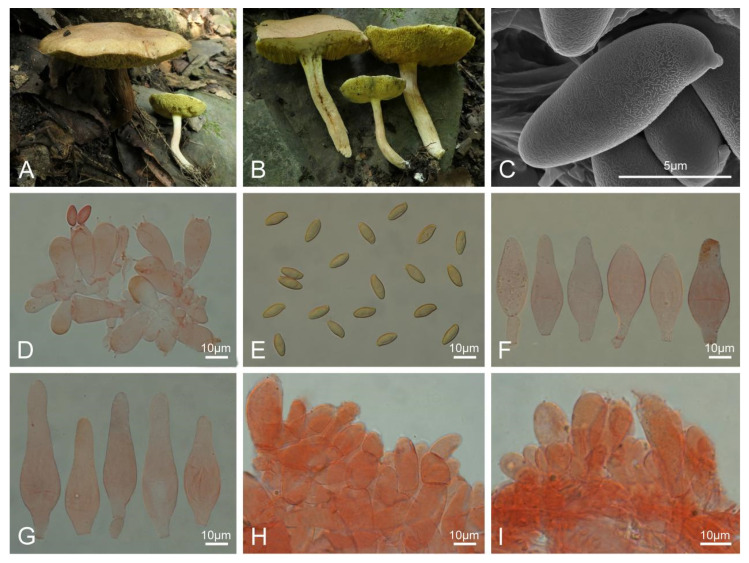
Basidiomata and microscopic features of *Xerocomus zhangii* (MHKMU L.J. Su 225, holotype). (**A**,**B**) Basidiomata. (**C**) Basidiospores under SEM. (**D**) Basidia. (**E**) Basidiospores. (**F**) Cheilocystidia. (**G**) Pleurocystidia. (**H**) Pileipellis. (**I**) Stipitipellis. (**A**–**C**) Photos by L.J. Su; (**D**–**K**) photos by R. Xue.

***Xerocomus zhangii*** L.P. Tang, R. Xue & L.J. Su sp. nov. (Figure 5).

MycoBank: MB 851817.

Chinese Name: 张氏绒盖牛肝菌

Etymology: Latin “*zhangii*”; is named after the family name of the selfless senior Mr. Chengyu Zhang (张澄宇), in appreciation of his help in collecting specimens in Anhui Province, as well as his contributions to and efforts in the development of the wild mushroom industry in Anhui and Yunnan Provinces.

Diagnosis: Differs from other species by a light brown to yellowish brown pileus, cyanescent context and hymenophore, a pale yellow to reddish stipe covered with longitudinal stripes.

Typification: CHINA. Anhui Province: Shitai Prefecture (石台县), Qidu Town (七都镇), Xiaohekou Village (小河口村), elev. 190 m, 3 August 2022, L.J. Su 225 (MHKMU L.J. Su 225, holotype). GenBank: 28S = PP179423; ITS = PP189879; *tef1* = PP230533; *rpb2* = PP195258.

*Basidiomata* small- to medium-sized. *Pileus* up to 6 cm diam., applanate; surface dry, velvety, grayish yellow (1B4), slightly reddish (5C4); context to 0.8 cm thick in the center of pileus, yellowish white (1A2), turning blue slowly, then fading later when injured. *Hymenophore* poroid, depressed around apex of stipe; pores 1 mm diam., angular to irregular, olive yellow (2C7), turning blue when injured; tubes up to 1 cm in length, concolorous with pores, turning blue when injured. *Stipe* 6.5 × 0.9 cm, central, subcylindrical, solid; surface dry, pale yellow (1A2) to reddish (5C4), covered with longitudinal stripes; context pale yellow (1A2) to reddish (5C4), turning blue when injured near the apex; annulus absent; basal mycelium white (1A1). *Odor* fragrant.

*Basidia* 20–33.5 × 8–12.5 μm, clavate, thin-walled, 4-spored, colorless to yellowish in KOH, occasionally with yellow pigments in the upper half; sterigmata 3–5 μm in length. *Basidiospores* [60/3/2] (8.5–)9–11.5(–12) × 4–5 μm, Q = 2.00–2.44(–2.63), Qm = 2.26 ± 0.14, yellowish to yellow in KOH, subfusiform to ellipsoid, with bacillate surface ornamentation under SEM. *Hymenophoral trama* phylloporoid, composed of hyphae 3.5–14.5 μm wide, colorless in KOH. *Cheilocystidia* 34–50 × 10.5–18 μm, abundant, fusiform to subfusiform, thin-walled, colorless in KOH, occasionally with yellow encrustations in the upper half. *Pleurocystidia* 45.5–71 × 10.5–19.5 μm, abundant, subfusiform or fusiform, thin-walled, colorless in KOH, occasionally with yellow encrustations in the upper half. *Pileipellis* epithelioid type, about 450 μm in thickness, composed of chains of subglobose to broadly subcylindrical cells up to 17 μm, thin- to slightly thick-walled (up to 0.5 μm), colorless to light yellow in KOH; terminal cells 13–46.5 × 8–17 μm, subpyriform, clavate to subglobose, with obtuse apex. *Pileus trama* composed of interlaced, branched, filamentous hyphae 7.5–14.5 μm wide, subcylindrical, thin- to slightly thick-walled (up to 0.5 μm), colorless in KOH. *Stipitipellis* hymeniform, about 200 μm in thickness, terminal cells 14–35 × 6.5–11 μm, subclavate to subfusiform, light yellow to yellow in KOH, and with clavate, four-spored basidia. *Stipe trama* composed of longitudinally arranged, parallel hyphae 5–17 μm diam., cylindrical, thin- to slightly thick-walled (up to 0.5 μm), colorless in KOH. *Clamp connections* absent in all tissues.

Habitat: Solitary on the ground in broad-leaved forests dominated by Fagaceae trees, e.g., *Cyclobalanopsis glauca*, *Castanea seguinii*, *Lithocarpus brevicaudatus*, and *Liquidambar formosana*.

Known distribution: Currently only known in Anhui Province (elevation about 200 m), eastern China.

Other specimens examined: CHINA. Anhui Province: Shitai Prefecture (石台县), Qidu Town (七都镇), Xiaohekou Village (小河口村), elev. 190 m, 3 August 2022, L.J. Su 225-1 (MHKMU L.J. Su 225-1).

Note*s: Xerocomus zhangii* is both morphologically similar and molecularly related to *X. fulvipes* and *X. galbanus*. However, *X. fulvipes* has a pale yellow-brown to pale red-brown pileus, a reddish brown stipe and narrower cystidia (35–72 × 8–14 μm) [36]; *X. galbanus* has a grayish white to pale yellow-brown pileus, non-cyanescent context, and larger basidiospores measuring 13–15 × 4.5–6 μm [79].

*Xerocomus zhangii* is also similar to *X. albotomentosus* N.K. Zeng, H.J. Xie, Chang Xu & Zhi Q. Liang, *X. fraternus* Xue T. Zhu & Zhu L. Yang, *X. rugosellus* (W.F. Chiu) F.L. Tai, and *X. subtomentosus* (L.) Quél. However, *X. albotomentosus* has a yellowish brown to dark brown pileus, non-cyanescent context, and a stipe base covered with obvious white villous mycelia [42]; *X. fraternus* has a yellowish brown to dull brown pileus, and a stipe without reddish tinge [36]; *X. rugosellus* has a green yellow hymenophore, non-cyanescent pores and context [15]; *X. subtomentosus* has a larger basidioma (up to 15 cm), brownish yellow to greenish yellow pores when aged, and larger basidiospores measuring 9.8–14.8 × 3.9–6 μm [85].

## 4. Discussion

Boletes are widely distributed in the subtropical and tropical regions of China. In the present study, we propose four new species, a new combination, and treat *Nigroboletus* as a synonym for *Xerocomellus* based on a comprehensive analysis of morphology, molecular biology, habitat, and host relationships.

*Xerocomus fulvipes* (Holotype HKAS68246) was originally described from Yunnan Province and also reported from Henan Province by Wu et al. in 2016 [36]. However, HKAS52556 from Yunnan Province was mislabeled as the holotype of this species in the molecular tree [36], and no sequence of the type specimen for this species could be found in GenBank. Recently, a new species, *X. galbanus,* described from Shanxi Province, was found to be closely related to *X. fulvipes* (HKAS52556) [79]. Interestingly, the specimen (HKAS76666, Henan Province) previously identified as *X. fulvipes* by Wu et al. in 2016 [36] clusters with the type specimen of *X. galbanus* in our molecular tree. Therefore, according to the current data, *X. fulvipes* is distributed only in Yunnan Province, southwestern China, while *X. galbanus* is distributed in Henan and Shanxi Provinces, central and northern China.

*Xerocomellus* was originally erected to accommodate *Xer. chrysenteron* and its relatives in 2008 [86]. The majority of species in this genus are distributed in North America and Europe [26,87,88,89], whereas just two species have been identified in China, viz. *Xer. communis* Xue T. Zhu & Zhu L. Yang and *Xer. corneri* [36]. In the present study, two new species of *Xerocomellus* were discovered in Anhui and Yunnan Provinces, increasing the species diversity of the genus in China.

*Nigroboletus* was proposed to accommodate *N. roseonigrescens*, a species described from tropical China [78]. Later, Farid et al. [77] built a relatively well-developed molecular tree comprising a wider range of species and showed that *Nigroboletus* is strongly supported as the base of all *Xerocomellus* sequences. Furthermore, our phylogenetic analysis indicates that *Nigroboletus* is embedded in the *Xerocomellus* lineage (Figure 1). Morphologically, *Nigroboletus* also shares some common characteristics with *Xerocomellus*, such as discoloration-prone basidiomata, velvety pileus surface, yellow-tinted pores and context, and the absence of reticulation on the stipe surface. Thus, we propose that *Nigroboletus* is improper as a separate genus and that it should be a synonym of *Xerocomellus*.

Sometimes, simple molecular phylogenetic analyses may reveal the wrong phylogenetic position of species, and similar faults have also occurred in other fungal groups. Shen et al. [90] conducted a systematic study of the genus *Hyphodermella* J. Erikss. & Ryvarden and suggested that as many integrated taxa as possible should be sampled for molecular phylogenetic analyses to avoid data limitations, especially when proposing new monotypic families or genera.

## Data Availability

The data obtained in this study have been publicly uploaded to NCBI GenBank, Treebase, and MycoBank.

## Supplementary Materials

The following are available online at: https://www.mdpi.com/article/10.3390/jof10050348/s1, Supplementary PDF S1: Figure S1. Phylogenetic tree of *Boletaceae* based on 28S dataset; Figure S2. Phylogenetic tree of *Boletaceae* based on *tef1* dataset; Figure S3. Phylogenetic tree of *Boletaceae* based on *rpb1* dataset; Figure S4. Phylogenetic tree of *Boletaceae* based on *rpb2* dataset.
