# Supplementary material for: Four New Species and a New Combination of Boletaceae (Boletales) from Subtropical and Tropical China
Source: J Fungi (Basel). 2024 May 13;10(5):348. doi: 10.3390/jof10050348 (PMC11122390; doi:10.3390/jof10050348)
Supplement: Supplementary file 1 [file jof-10-00348-s001.zip › jof-2972383-supplementary.pdf]

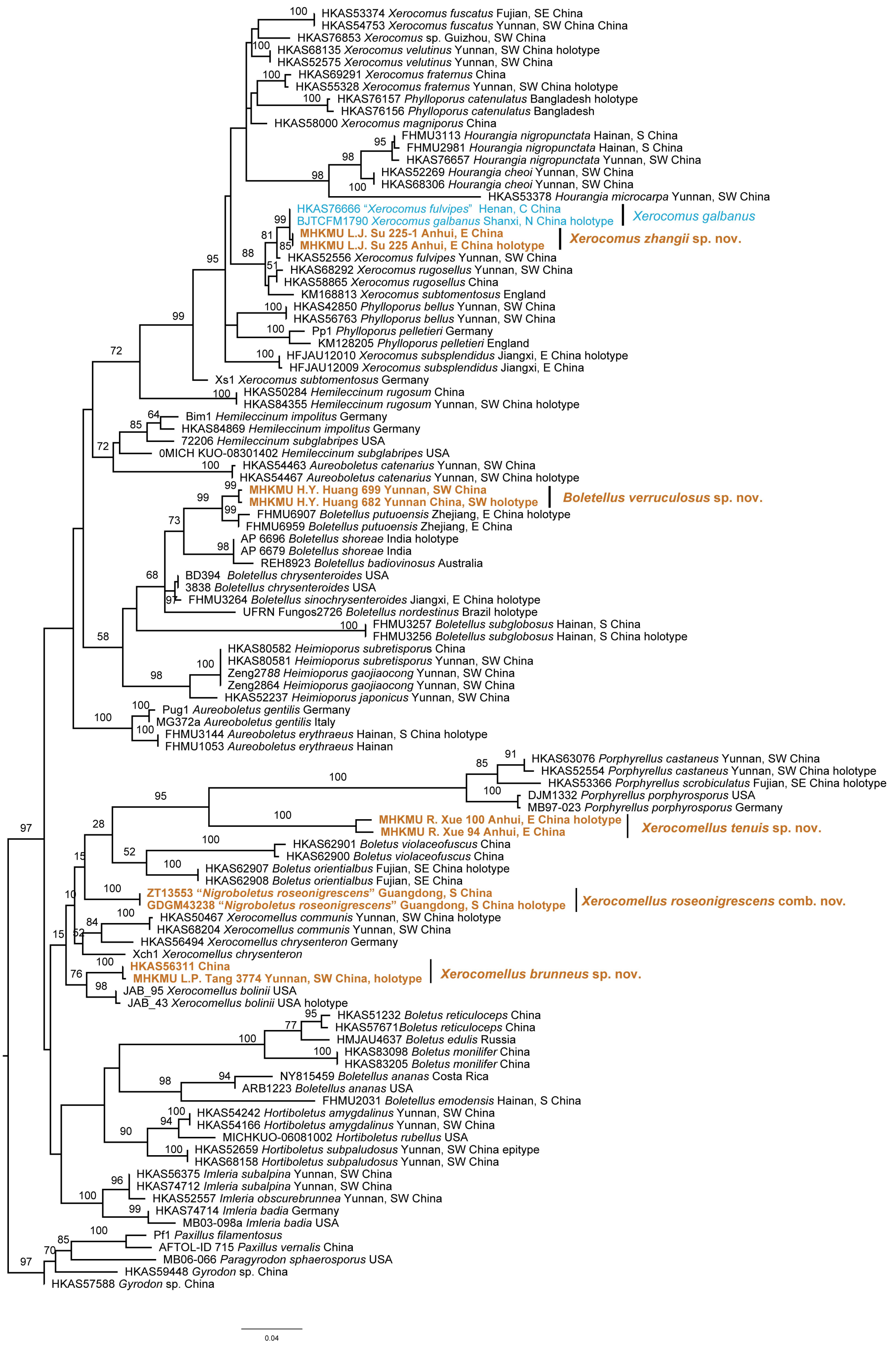


**Figure S1.** Phylogenetic tree of *Boletaceae* based on 28S dataset. RAxML BP values (≥50%) are shown above branches. Notes: C = Central, E = Eastern, N = Northern, S = Southern, SE = Southeastern, SW = Southwestern.

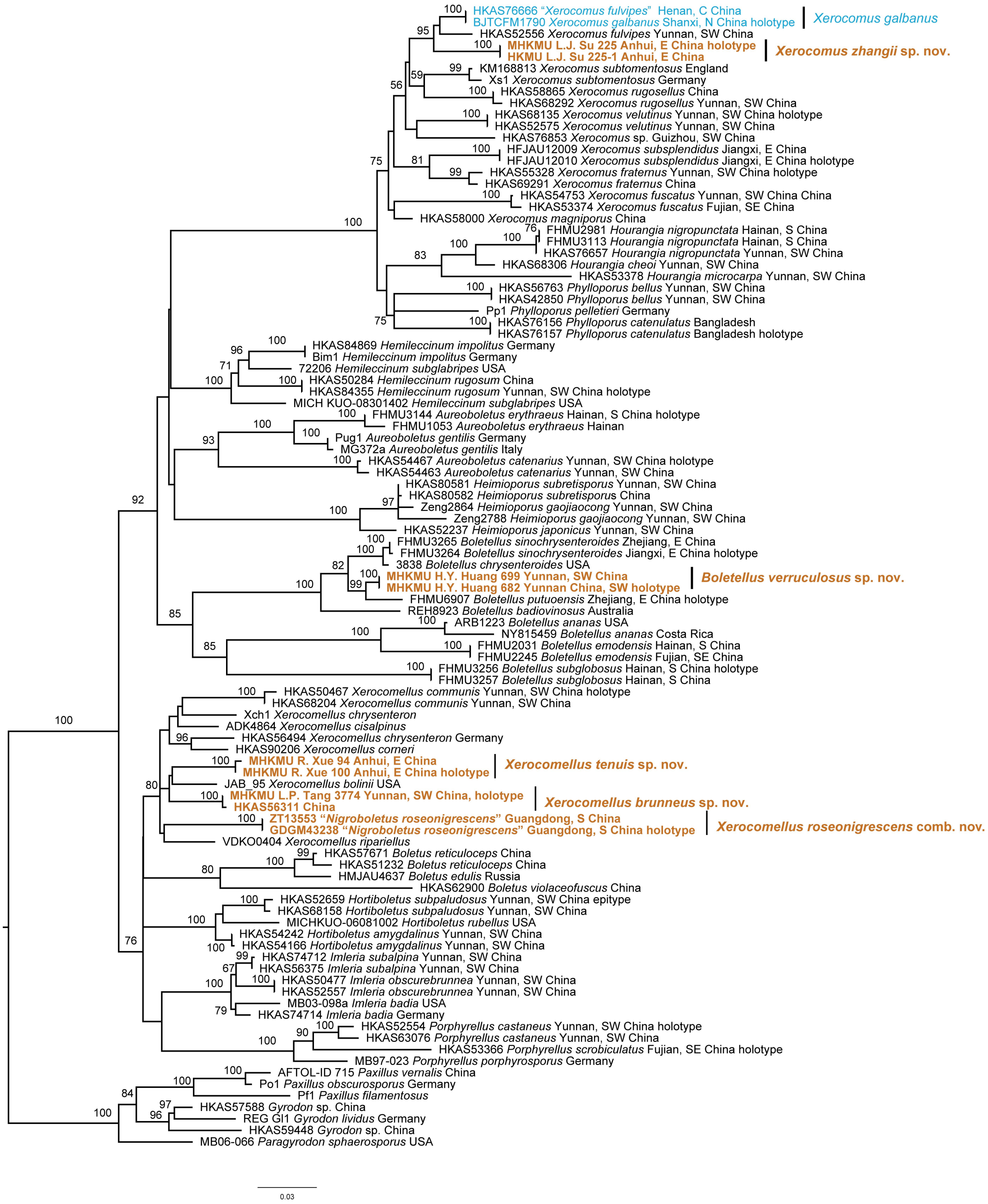


**Figure S2.** Phylogenetic tree of *Boletaceae* based on *tef1* dataset. RAxML BP values (≥50%) are shown above branches. Notes: C = Central, E = Eastern, N = Northern, S = Southern, SE = Southeastern, SW = Southwestern.

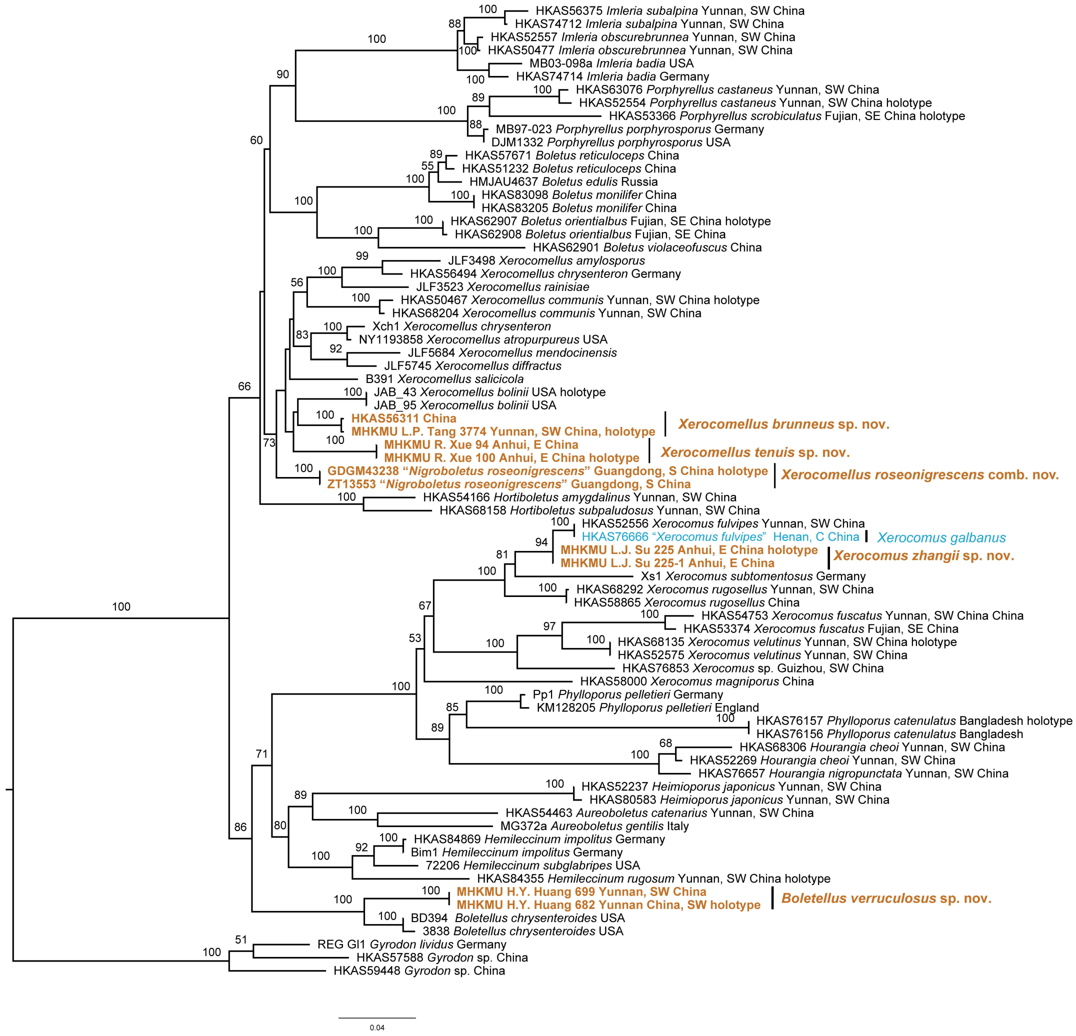


**Figure S3.** Phylogenetic tree of *Boletaceae* based on *rpb1* dataset. RAxML BP values (≥50%) are shown above branches. Notes: C = Central, E = Eastern, N = Northern, S = Southern, SE = Southeastern, SW = Southwestern.

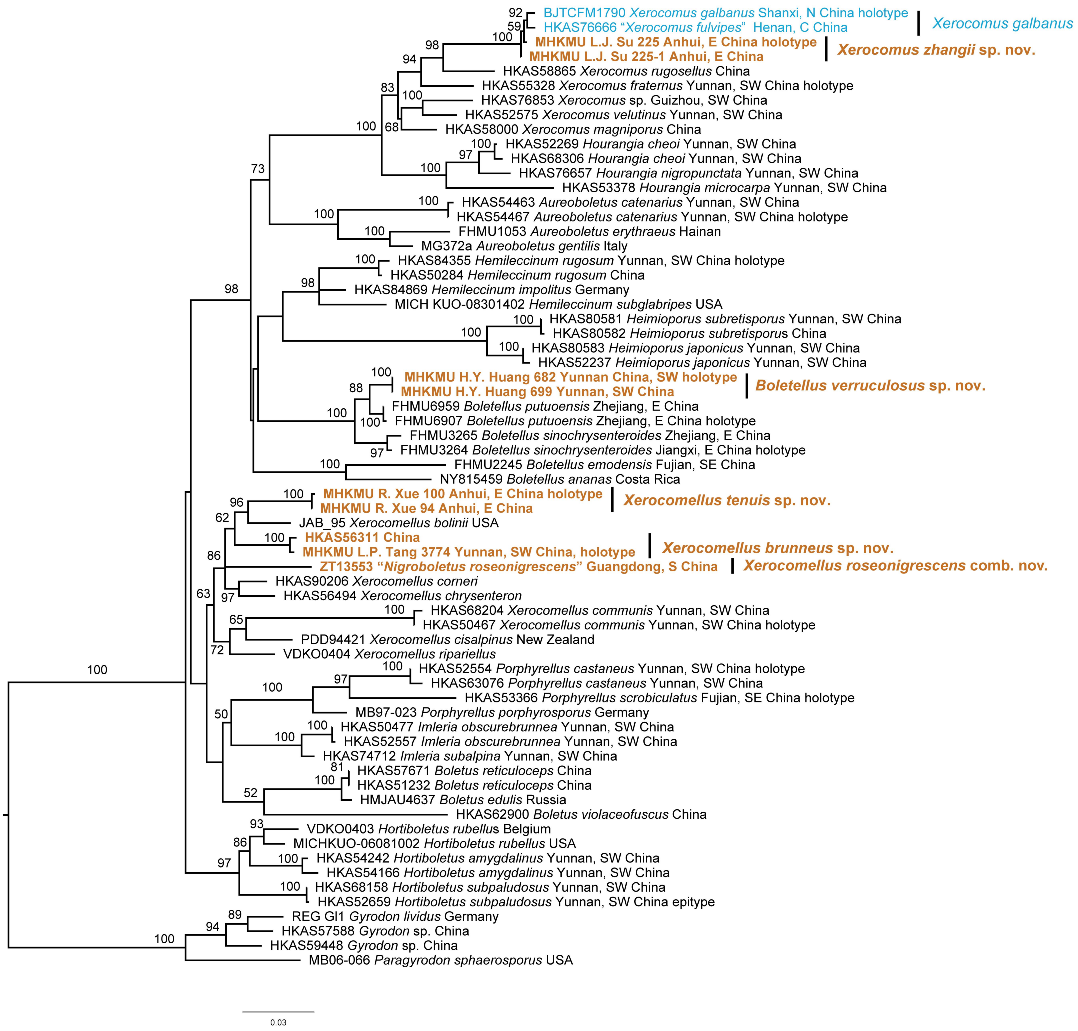


**Figure S4.** Phylogenetic tree of *Boletaceae* based on *rpb2* dataset. RAxML BP values (≥50%) are shown above branches. Notes: C = Central, E = Eastern, N = Northern, S = Southern, SE = Southeastern, SW = Southwestern.
